# Supplementary material for: Proposal of a Mathematical Model to Monitor Body Mass over Time in Subjects on a Diet
Source: Nutrients. 2022 Aug 30;14(17):3575. doi: 10.3390/nu14173575 (PMC9460375; doi:10.3390/nu14173575)
Supplement: Supplementary file 1 [file nutrients-14-03575-s001.zip › nutrients-1849269-supplementary.pdf]

nutrients

MDPI

Supplementary Table

Table S1. Body Mass fitting time course

Sex	AGE [years]	Initial Mass (M_i) [kg]	BMI [kg/m²]	Initial Km (K_{mi}) [h⁻¹]	Final Mass (M_f) [kg]	Final Km (K_{mf}) [h⁻¹]	DF [n-1]	Dev	Var
F	17	60.0	20.0	0.000918	57.8	0.000949	5	3.030	0.606
F	47	74.0	27.5	0.001415	69.8	0.001165	4	0.330	0.083
F	54	93.8	35.3	0.000928	78.4	0.000941	6	1.180	0.197
F	65	73.0	31.2	0.001469	63.6	0.001592	4	0.010	0.003
F	55	68.2	26.0	0.001278	64.0	0.001205	4	0.020	0.005
F	18	55.6	21.2	0.001027	54.6	0.000967	4	0.040	0.010
M	65	93.0	32.2	0.000768	80.2	0.001019	12	4.200	0.350
F	67	102.0	37.9	0.001123	94.2	0.000866	5	2.090	0.418
F	25	103.2	38.4	0.000646	101.6	0.000677	4	0.180	0.045
F	23	57.8	23.4	0.001452	50.8	0.001355	5	1.150	0.230
F	40	56.2	21.7	0.001519	52.2	0.001700	4	0.340	0.085
F	25	102.4	38.1	0.000862	80.0	0.000751	9	4.800	0.533
F	46	66.8	28.2	0.001987	61.8	0.001659	7	2.650	0.379
F	34	81.4	25.4	0.001050	71.0	0.000996	7	1.550	0.221
F	46	73.8	28.5	0.001034	66.6	0.000977	5	0.480	0.096
F	69	83.8	33.4	0.000720	67.6	0.000832	5	1.050	0.210
F	28	54.6	20.8	0.001604	49.0	0.001720	5	0.020	0.004
F	32	63.3	24.1	0.001856	60.1	0.001637	7	1.860	0.266
F	60	69.2	27.4	0.001130	65.4	0.001046	5	4.630	0.926
F	32	62.9	26.5	0.001014	55.6	0.000935	5	1.200	0.240
F	17	65.6	25.9	0.001186	60.4	0.001219	4	0.200	0.050
M	30	87.4	29.5	0.001826	69.0	0.001784	4	4.490	1.123
F	37	104.8	39.9	0.001428	91.0	0.001018	4	0.450	0.113

M	12	40.0	21.0	0.001596	35.6	0.001626	4	0.020	0.005
M	49	91.6	32.5	0.001385	82.4	0.001091	4	0.050	0.013
F	16	68.6	22.7	0.001289	64.4	0.001290	13	3.470	0.267
F	32	58.0	20.3	0.001143	66.0	0.001125	8	0.920	0.115
F	20	35.8	12.9	0.002939	40.4	0.003350	9	9.020	1.002
F	34	99.8	38.5	0.000656	88.9	0.000602	8	4.900	0.613
F	67	79.5	29.9	0.000658	73.0	0.000664	5	0.510	0.102
F	50	53.9	23.3	0.002167	47.0	0.002352	4	0.340	0.085
F	32	80.2	27.8	0.002369	74.0	0.002020	5	0.590	0.118
F	34	58.0	22.9	0.002002	54.6	0.001903	4	4.590	1.148
M	37	91.9	27.1	0.001151	87.2	0.000985	5	1.900	0.380
F	25	56.4	22.0	0.001239	51.5	0.001072	7	7.360	1.051
F	20	58.4	21.8	0.001191	48.0	0.001831	4	0.040	0.010
F	25	74.2	29.0	0.002323	65.0	0.002034	8	12.520	1.565
F	29	64.9	24.7	0.002169	60.3	0.001950	6	0.940	0.157
F	35	76.8	30.0	0.001021	63.5	0.000993	5	0.620	0.124
F	31	54.2	24.1	0.001751	48.6	0.001733	4	0.520	0.130
F	35	84.4	32.9	0.001070	73.6	0.001074	4	1.320	0.330
M	41	76.2	25.5	0.001412	68.8	0.001546	5	0.100	0.020
F	56	73.3	29.4	0.000999	65.6	0.000983	5	1.240	0.248
F	52	68.7	24.9	0.000714	62.3	0.000000	4	0.380	0.095
F	28	97.8	31.9	0.000964	87.4	0.000957	7	4.370	0.624
M	52	78.0	28.7	0.001388	65.0	0.001321	4	0.440	0.110
F	36	68.6	29.3	0.001539	55.6	0.001522	7	0.460	0.066
F	27	52.2	17.4	0.001578	54.9	0.001586	5	0.050	0.010
M	41	76.0	29.7	0.001835	77.6	0.001851	7	0.660	0.094
F	32	71.8	24.0	0.001807	63.2	0.001951	4	1.030	0.258
F	35	70.6	27.7	0.001052	63.6	0.001082	5	0.580	0.116
F	48	79.2	37.2	0.001184	64.0	0.001348	5	0.180	0.036
F	26	99.3	33.2	0.001389	71.2	0.001360	14	1.440	0.103

F	28	75.2	25.1	0.001466	86.3	0.001366	6	4.690	0.782
F	25	72.4	24.4	0.000761	68.2	0.000689	6	0.410	0.068
M	38	85.2	26.0	0.001494	73.6	0.001417	4	0.620	0.155
F	42	61.0	22.4	0.001935	56.2	0.002000	4	0.040	0.010
F	55	103.2	39.4	0.001076	85.2	0.001066	9	4.860	0.540
F	62	66.4	29.4	0.001042	58.1	0.001095	5	0.580	0.116
F	57	85.5	32.6	0.000961	78.3	0.001064	4	1.060	0.265
F	53	77.0	32.1	0.000604	69.0	0.006340	6	0.150	0.025
F	51	73.4	28.0	0.001472	65.0	0.001409	4	0.990	0.248
F	39	55.0	22.6	0.001628	52.0	0.001737	4	0.140	0.035
F	29	89.9	32.7	0.001412	85.3	0.001395	6	0.750	0.125
F	37	60.0	23.1	0.001646	55.8	0.001631	4	0.030	0.008
F	65	71.2	29.6	0.001586	61.4	0.001612	12	1.690	0.141
F	27	61.0	21.1	0.001215	58.0	0.001190	5	0.630	0.126
F	39	74.8	30	0.001186	62.6	0.001351	4	1.110	0.278
F	60	63.8	27.5	0.001288	60.2	0.001439	4	0.500	0.125
F	28	59.2	26.3	0.001405	54.0	0.001672	4	0.640	0.160
M	33	117.1	44.7	0.001432	88.1	0.002131	5	0.380	0.076
F	34	71.6	26.5	0.001016	65.0	0.001046	4	5.720	1.430
F	26	65.2	27.8	0.001446	59.8	0.001578	5	0.300	0.060
F	28	58.6	23.1	0.003295	54.8	0.003595	4	0.650	0.163
M	25	83.2	27.7	0.001389	74.4	0.001195	4	0.000	0.000
M	29	73.0	25.5	0.001258	63.6	0.001258	5	0.110	0.022
M	26	117.4	36.2	0.001276	75.6	0.001303	9	2.120	0.236
F	24	62.6	22.7	0.001514	58.8	0.001477	4	0.090	0.023
M	65	125.8	41.9	0.000772	109.1	0.000628	5	0.800	0.160
F	31	79.0	28.9	0.001162	71.6	0.001305	7	8.950	1.279
F	42	86.9	34.3	0.000989	80.5	0.001060	8	19.420	2.428
F	37	103.8	44.4	0.000813	96.3	0.000794	4	0.360	0.090
F	43	57.6	26.7	0.001185	54.0	0.001340	7	1.720	0.246

F	58	74.9	26.2	0.001428	67.8	0.001480	4	0.040	0.010
M	38	88.8	27.7	0.001467	86.0	0.001443	4	0.160	0.040
F	49	62.7	32.5	0.001254	54.4	0.001471	4	0.120	0.030
M	33	93.6	29.8	0.001073	80.8	0.001053	4	4.240	1.060
F	28	76.4	27.4	0.001326	66.6	0.001299	4	0.820	0.205
F	22	50.0	16.8	0.002488	56.1	0.002282	5	1.790	0.358
F	25	75.2	32.6	0.001549	67.6	0.001627	7	2.950	0.421
F	24	68.4	26.1	0.001024	79.8	0.000914	5	5.430	1.086
F	23	85.0	32.8	0.001620	68.7	0.001599	5	0.680	0.136
F	46	82.4	33.0	0.001524	69.5	0.001767	8	18.250	2.281
F	37	71.9	29.9	0.000854	67.0	0.000846	7	10.100	1.443
F	52	70.4	27.1	0.000969	59.6	0.001160	4	0.000	0.000
F	27	61.3	22.6	0.001786	57.0	0.001733	4	1.810	0.453
F	22	75.2	32.8	0.001176	67.0	0.001203	5	0.490	0.098
F	44	74.8	29.1	0.001024	67.0	0.001004	9	60.840	6.760
F	61	73.0	27.8	0.001498	65.8	0.001462	5	0.110	0.022
M	62	117.8	33.6	0.000637	111.8	0.000625	5	0.980	0.196
F	37	68.5	27.5	0.001803	65.6	0.001792	4	0.300	0.075
F	37	73.0	36.5	0.001239	66.3	0.001186	4	0.000	0.000
F	42	74.0	26.2	0.001418	66.6	0.001466	5	3.890	0.778
F	27	56.0	23.0	0.001787	68.8	0.001430	4	0.220	0.055
F	26	60.4	26.3	0.001963	57.0	0.001918	4	0.070	0.018
F	38	90.8	33.7	0.000752	84.2	0.000580	4	0.290	0.073
F	56	100.2	41.1	0.001231	92.8	0.001169	4	0.390	0.098
F	35	69.4	26.8	0.000978	65.4	0.001075	4	0.710	0.178
F	36	78.0	26.9	0.001208	71.6	0.001164	7	4.540	0.649
F	48	89.8	36.4	0.000624	62.0	0.000600	5	5.300	1.060
F	36	83.0	29.7	0.001909	74.2	0.001824	6	2.230	0.372
F	29	77.3	29.8	0.002217	64.8	0.002694	6	1.790	0.298
M	51	116.4	35.5	0.001090	96.8	0.000999	6	2.240	0.373

F	25	83.6	33.2	0.001353	75.0	0.001198	4	1.320	0.330
F	45	75.2	29.0	0.001808	69.5	0.001748	4	1.430	0.358
M	70	83.8	30.4	0.000956	80.0	0.000885	5	0.450	0.090
F	66	71.8	27.0	0.000734	67.5	0.000700	4	0.570	0.143
F	58	70.2	30.4	0.001258	65.0	0.001240	4	0.120	0.030
M	53	70.8	25.1	0.001619	63.4	0.001520	4	0.180	0.045
F	37	72.4	25.0	0.001571	66.4	0.001523	5	1.920	0.384
F	19	95.9	36.0	0.001410	76.5	0.001813	11	5.100	0.464
F	48	100.3	38.1	0.001227	85.1	0.001205	7	7.550	1.079
M	35	104.2	33.2	0.001217	83.9	0.001310	9	3.670	0.408
F	27	94.0	35.3	0.001158	84.2	0.001312	6	0.680	0.113
F	39	72.2	29.8	0.001275	65.8	0.001275	5	0.290	0.058
F	27	56.8	25.2	0.001323	52.0	0.001295	9	0.880	0.098
F	28	71.2	26.3	0.001352	65.3	0.001375	6	0.690	0.115
F	56	54.8	21.7	0.001119	50.0	0.001105	4	0.360	0.090
F	51	79.6	31.1	0.001904	65.0	0.001821	5	0.380	0.076
F	57	61.2	25.4	0.002662	58.0	0.002823	5	1.100	0.220
F	40	92.6	36.9	0.001365	78.5	0.001173	4	0.000	0.000
F	29	64.2	23.0	0.001541	59.2	0.001666	5	0.000	0.000
F	57	66.4	25.0	0.001521	64.8	0.001536	5	0.740	0.148
F	31	64.0	26.7	0.000951	60.6	0.000885	5	1.990	0.398
F	31	98.5	33.7	0.001444	81.0	0.001618	7	6.490	0.927
M	28	83.8	26.4	0.001605	76.4	0.001786	5	4.330	0.866
M	62	82.0	31.2	0.001364	74.6	0.001345	4	0.140	0.035
M	61	65.4	24.9	0.001445	59.4	0.001599	4	0.570	0.143
F	53	64.5	24.8	0.001467	61.8	0.001440	4	0.770	0.193
M	29	99.5	30.4	0.000857	94.0	0.000841	5	4.620	0.924
F	53	70.6	30.1	0.001464	64.0	0.001544	5	0.480	0.096
F	59	94.8	38.5	0.000913	85.4	0.000894	5	0.630	0.126
F	32	66.4	26.3	0.001722	62.8	0.001996	4	0.030	0.008

F	25	62.6	24.7	0.001949	55.6	0.001835	4	0.940	0.235
F	52	59.2	21.7	0.002139	56.0	0.002177	4	2.960	0.740
M	24	79.8	26.7	0.001597	70.0	0.001938	6	3.240	0.540
F	33	67.8	25.8	0.001301	65.2	0.001346	5	0.640	0.128
F	34	72.8	29.2	0.003497	65.2	0.003552	5	2.680	0.536
F	28	69.4	26.12	0.001558	66.4	0.001581	4	0.070	0.018
F	49	98.6	36.6	0.000925	89.3	0.000912	4	0.010	0.003
F	52	84.9	35.8	0.000712	79.0	0.000716	4	0.190	0.048
F	56	109.0	43.6	0.000886	101.8	0.000832	4	0.350	0.088
F	35	56.0	20.6	0.000813	53.0	0.000811	6	1.420	0.237
F	48	78.8	31.9	0.001613	73.0	0.001589	5	2.990	0.598
M	40	98.6	30.7	0.001344	85.0	0.001271	4	0.190	0.048
F	37	53.2	21	0.000992	52.6	0.001031	5	0.620	0.124
F	39	92.0	37.8	0.001086	81.8	0.001067	5	0.320	0.064
F	35	69.8	26.9	0.000964	63.8	0.001067	4	0.240	0.060
F	56	61.2	25.5	0.001035	55.8	0.001377	5	1.130	0.226
F	27	59.8	20.7	0.002286	71.0	0.001497	6	17.020	2.837
F	28	95.2	36.7	0.001134	86.0	0.001074	6	2.930	0.488
F	25	79.8	28.3	0.000893	66.8	0.000830	6	1.010	0.168
F	23	74.1	26.6	0.000880	64.8	0.000876	4	0.030	0.008
F	24	68.3	27.4	0.001018	60.4	0.001000	9	5.310	0.590
F	25	60.4	24.2	0.000871	73.0	0.000713	6	5.660	0.943
F	27	71.2	29.2	0.001046	65.2	0.001179	4	1.680	0.420
F	36	75.2	28.6	0.002012	63.0	0.001939	4	0.000	0.000
F	52	117.4	39.7	0.001285	97.0	0.001225	5	1.180	0.236
F	41	86.0	36.3	0.000953	79.0	0.000979	7	1.320	0.189
F	37	68.0	26.6	0.001301	58.4	0.001449	5	0.430	0.086
F	26	56.8	21.1	0.001780	53.0	0.001760	4	0.240	0.060
F	27	53.7	20	0.001553	63.5	0.001312	6	2.250	0.375
M	26	90.0	33.5	0.001260	78.6	0.001128	5	2.290	0.458

F	35	64.8	24.7	0.001725	60.5	0.001712	4	0.350	0.088
F	36	71.0	26.7	0.001718	64.4	0.001923	5	3.090	0.618
M	50	84.0	29.4	0.001303	79.3	0.001333	4	1.120	0.280
F	44	78.0	27.3	0.001040	70.8	0.000974	5	1.700	0.340
F	56	90.2	30.7	0.001904	79.5	0.002011	10	2.960	0.296
F	34	82.5	31.9	0.000986	72.9	0.000971	5	0.530	0.106
F	36	60.8	21.8	0.001504	58.6	0.001462	4	0.390	0.098
F	27	48.8	18.8	0.001293	51.0	0.001305	4	0.020	0.005
F	28	50.4	19.4	0.001655	61.5	0.000834	4	0.160	0.040
F	57	60.2	22.4	0.001282	56.8	0.001373	4	0.530	0.133
M	38	78.6	27.2	0.001163	70.8	0.001168	6	2.270	0.378
F	48	82.0	32	0.001051	67.4	0.000996	5	0.020	0.004
M	27	82.2	25.6	0.001213	72.6	0.001212	7	0.030	0.004
F	39	70.0	23.4	0.001671	66.1	0.001703	5	1.010	0.202
F	38	73.2	23.9	0.002141	67.2	0.002088	4	1.070	0.268
F	42	53.0	22.1	0.001904	51.2	0.002022	4	0.470	0.118
F	32	84.5	30.3	0.001107	72.3	0.001072	6	2.190	0.365
M	53	115.0	37.1	0.001004	105.8	0.000965	4	0.000	0.000
F	44	76.0	29.3	0.001418	67.5	0.001511	5	3.310	0.662
M	61	67.2	26.6	0.001079	62.4	0.001043	5	0.460	0.092
F	53	91.0	33.4	0.000972	72.0	0.000932	4	0.330	0.083
F	26	99.0	35.1	0.000955	84.4	0.001164	8	9.090	1.136
F	37	54.2	21.7	0.001092	51.9	0.001309	5	0.390	0.078
M	41	94.6	34.7	0.001158	86.4	0.001098	4	0.540	0.135
F	55	66.6	26.6	0.001671	62.0	0.001573	4	0.180	0.045
F	47	71.6	27.6	0.001082	64.8	0.001084	4	0.310	0.078
F	27	75.4	29.1	0.001531	60.2	0.001500	7	0.940	0.134
F	28	58.2	20.1	0.001518	70.6	0.000913	7	14.390	2.056
F	33	67.2	23.8	0.001612	62.0	0.001618	4	0.210	0.053
F	38	106.2	33	0.000997	86.6	0.000984	7	3.800	0.543

F	48	99.6	35.3	0.001528	84.8	0.002029	5	4.540	0.908
F	55	67.2	25.3	0.001784	61.8	0.001871	4	0.160	0.040
M	13	98.3	39.4	0.001043	92.0	0.000993	8	3.200	0.400
F	37	66.0	26.1	0.000891	58.0	0.000918	5	0.650	0.130
M	48	90.4	28.2	0.002129	75.8	0.002005	4	0.530	0.133
F	42	98.5	38.9	0.000516	88.0	0.000466	7	1.120	0.160
F	58	78.8	32.4	0.001347	69.8	0.001470	7	1.250	0.179
F	42	65.8	26	0.002239	56.3	0.002199	4	0.350	0.088
F	43	60.8	26.7	0.001495	55.8	0.001612	4	0.010	0.003
F	26	96.6	38.3	0.000847	91.8	0.000845	7	4.120	0.589
F	46	94.4	38.2	0.000873	80.6	0.000978	7	0.420	0.060
F	64	95.0	35.7	0.001354	86.2	0.001387	8	2.920	0.365
M	73	95.2	34.9	0.000698	91.8	0.000765	4	2.930	0.733
F	36	62.8	25.1	0.001312	59.4	0.001277	7	1.990	0.284
F	42	46.2	18.5	0.000921	48.0	0.000964	8	0.920	0.115
F	64	85.8	33.5	0.001283	77.6	0.001379	4	1.610	0.403
F	57	84.5	30.1	0.001132	73.2	0.001013	5	0.370	0.074
F	24	68.0	29.9	0.001621	61.9	0.001651	4	0.370	0.093
F	38	72.0	29.5	0.001418	66.6	0.001386	5	0.990	0.198
F	29	66.3	27.2	0.001141	57.9	0.001041	4	0.250	0.063
F	25	82.3	27.8	0.001361	77.5	0.001317	4	0.030	0.008
M	46	105.6	30.5	0.000769	91.8	0.000998	5	2.550	0.510
F	23	61.8	22.7	0.001706	56.6	0.002015	4	0.260	0.065
M	19	106.2	34.2	0.001826	92.2	0.001692	10	10.050	1.005
F	29	93.4	39.5	0.000758	86.0	0.000722	4	1.690	0.423
M	18	74.6	24.9	0.001316	67.8	0.001250	4	1.460	0.365
M	46	88.7	31.4	0.000759	73.3	0.000810	5	1.310	0.262
F	20	58.2	23.6	0.001460	52.2	0.001573	4	0.420	0.105
F	47	65.0	27	0.001378	59.2	0.001461	4	2.040	0.510
F	30	74.8	31.9	0.001060	64.1	0.001006	4	0.080	0.020

F	56	82.5	29.6	0.000737	78.2	0.000682	4	0.020	0.005
F	28	59.6	23.3	0.001227	55.1	0.001183	5	1.830	0.366
F	29	63.1	24.6	0.001021	72.7	0.000864	5	1.900	0.380
F	43	82.8	35.8	0.001090	66.8	0.001212	4	0.000	0.000
F	55	84.3	32.5	0.000975	73.6	0.001101	5	1.440	0.288
F	57	63.0	25.9	0.000821	59.3	0.000813	4	0.070	0.018
F	62	86.8	36.1	0.000765	68.3	0.000854	4	0.010	0.003
F	58	66.0	25.5	0.001435	60.6	0.001429	5	3.190	0.638
F	26	74.8	25.3	0.001189	69.0	0.001216	5	3.160	0.632
F	31	76.1	29.3	0.001221	65.6	0.001293	4	0.300	0.075
F	28	80.6	31.1	0.000869	59.2	0.001002	7	3.390	0.484
F	17	70.5	29.3	0.000811	65.6	0.000820	4	0.020	0.005
M	19	67.0	21.6	0.001142	64.2	0.001184	5	1.470	0.294
F	26	64.0	24.1	0.001381	78.2	0.001246	6	4.560	0.760
F	48	91.1	34.7	0.001183	81.6	0.001044	4	0.000	0.000
F	25	74.8	31.5	0.001093	81.0	0.001392	7	3.520	0.503
F	41	57.5	25.2	0.001545	55.4	0.001369	6	0.450	0.075
F	34	60.8	22.3	0.001680	52.6	0.001300	5	7.400	1.480
F	31	53.8	21.3	0.002126	55.0	0.002621	4	0.000	0.000
F	52	83.9	34.0	0.000733	74.5	0.000806	6	2.000	0.333
F	35	65.9	23.6	0.001136	72.9	0.001223	8	0.810	0.101
F	53	60.0	22.6	0.001387	53.3	0.001786	5	0.030	0.006
F	67	84.4	37.0	0.001120	75.1	0.001388	6	0.730	0.122
F	46	74.8	29.6	0.001240	67.0	0.001061	5	0.150	0.030
M	40	95.2	30.7	0.001473	83.0	0.001382	6	3.240	0.540
F	33	63.2	25.0	0.001164	53.1	0.001000	4	0.020	0.005
F	25	65.2	25.2	0.001762	59.4	0.001640	5	0.420	0.084
F	38	49.8	19.0	0.001771	52.0	0.001718	5	0.590	0.118
F	63	82.0	29.8	0.001420	71.0	0.001458	6	1.370	0.228
F	59	67.8	28.6	0.001303	57.8	0.001071	5	0.120	0.024

F	40	77.4	31.4	0.000897	75.5	0.000959	4	0.000	0.000
F	43	56.0	21.9	0.001476	52.2	0.001647	4	0.060	0.015
F	35	63.4	22.5	0.001151	60.2	0.001204	6	0.840	0.140
M	23	127.8	37.3	0.000905	109.0	0.000673	6	4.490	0.748
F	70	91.5	32.4	0.001164	80.0	0.001306	4	0.030	0.008
F	41	68.8	27.2	0.001799	65.0	0.001747	6	0.720	0.120
F	67	85.1	37.8	0.000751	80.5	0.000721	5	3.220	0.644
F	25	67.8	22.5	0.001536	66.6	0.001601	7	4.700	0.671
F	34	69.4	25.5	0.001167	64.0	0.001119	5	0.890	0.178
F	19	83.4	33.4	0.001199	75.1	0.000906	4	0.370	0.093
F	35	91.1	33.5	0.001117	85.6	0.000974	8	1.290	0.161
F	69	72.0	31.2	0.001047	67.0	0.000933	5	0.240	0.048
F	47	74.6	27.7	0.001302	70.6	0.001062	4	0.070	0.018
F	25	56.0	22.2	0.002212	51.6	0.002299	5	0.230	0.046
F	22	84.8	36.7	0.000479	68.8	0.000395	5	2.820	0.564
F	24	77.8	31.6	0.000977	73.0	0.000938	4	0.210	0.053
F	48	75.7	26.4	0.001173	69.4	0.001236	6	25.820	4.303
M	49	94.9	29.1	0.001362	86.8	0.001130	4	0.010	0.003
F	38	60.6	21.5	0.001586	72.1	0.001202	8	0.470	0.059
F	47	76.6	31.5	0.000986	67.0	0.000764	6	0.980	0.163
F	53	96.3	37.2	0.000853	86.2	0.007850	5	1.490	0.298
F	41	67.6	28.5	0.002109	62.8	0.002047	4	0.140	0.035
F	51	61.8	26.1	0.001152	58.0	0.001089	6	0.630	0.105
M	19	135.0	41.7	0.001006	125.0	0.000815	6	1.250	0.208
F	37	64.4	25.5	0.001417	59.4	0.001371	5	1.740	0.348
F	51	78.8	40.2	0.000977	74.2	0.000909	6	0.520	0.087
F	52	100.3	38.2	0.001269	85.1	0.000766	7	3.730	0.533
F	54	84.3	32.9	0.001125	82.3	0.000842	5	3.380	0.676
F	33	108.7	41.9	0.000838	83.5	0.000673	8	2.610	0.326
F	52	55.8	21.3	0.001885	54.0	0.000989	4	0.570	0.143

M	56	74.4	25.1	0.002010	67.5	0.001687	5	4.240	0.848
F	25	47.2	17.8	0.001562	46.6	0.001512	4	0.150	0.038
F	56	75.4	31	0.001529	69.0	0.001293	5	1.540	0.308
F	13	70.6	24.8	0.002235	66.2	0.001884	6	0.390	0.065
F	15	80.2	25.0	0.001253	73.0	0.001092	4	3.070	0.768
F	64	74.5	31.0	0.001021	59.0	0.000917	5	0.190	0.038
M	63	77.8	26.3	0.001153	71.2	0.001487	5	0.030	0.006
F	44	77.0	26.6	0.001424	70.2	0.001236	6	0.420	0.070
F	44	56.4	23.2	0.001804	55.2	0.001648	6	0.390	0.065
F	13	50.2	25.4	0.001143	48.4	0.001203	5	0.540	0.108
M	42	105.0	29.1	0.001168	90.6	0.001038	5	0.680	0.136
F	19	49.2	19.5	0.001598	48.0	0.001779	7	1.210	0.173
F	25	55.0	23.0	0.001539	50.0	0.001434	4	0.190	0.048
F	58	106.3	34.3	0.001793	86.1	0.001470	4	0.140	0.035
F	71	57.0	21.5	0.001962	50.0	0.002341	7	3.370	0.481
F	50	66.0	27.1	0.000913	62.0	0.000818	5	2.010	0.402
F	25	46.8	17.2	0.001667	49.6	0.001684	5	1.070	0.214
F	7	34.0	22.5	0.002111	34.8	0.002043	5	0.420	0.084
F	58	53.8	24.2	0.001197	47.8	0.001180	4	0.070	0.018
F	64	82.7	29.0	0.000749	79.0	0.000722	5	3.120	0.624
F	60	66.4	25.9	0.001716	61.4	0.001394	6	1.110	0.185
F	59	87.4	36.9	0.000751	74.1	0.000722	6	0.540	0.090
F	26	61.0	22.1	0.001085	53.8	0.001131	9	5.700	0.633
F	15	61.4	26.6	0.001141	57.0	0.001034	5	0.430	0.086
F	71	68.2	26.6	0.001181	58.9	0.001085	4	0.000	0.000
F	54	71.3	31.3	0.001308	60.9	0.001237	5	0.030	0.006
F	46	69.6	25.6	0.000823	55.5	0.000881	4	0.300	0.075
F	16	75.1	26.9	0.000822	70.0	0.000816	4	0.380	0.095
M	41	81.3	29.5	0.001093	72.9	0.000928	4	0.120	0.030
F	18	73.7	28.6	0.001790	63.2	0.001703	5	1.960	0.392

F	35	72.2	28.2	0.001241	65.6	0.001225	4	0.040	0.010
F	64	53.4	22.1	0.002422	49.8	0.002560	8	1.820	0.228
F	31	97.0	38.9	0.001093	97.0	0.000879	10	2.890	0.289
F	32	66.4	26.3	0.001335	58.9	0.001278	5	0.000	0.000
F	14	42.8	15.3	0.002320	49.8	0.002194	6	4.230	0.705
F	55	80.0	31.2	0.001112	70.2	0.000966	5	0.370	0.074
M	25	119.2	41.2	0.000774	83.1	0.001094	8	0.220	0.028
F	47	75.0	29.7	0.001225	71.5	0.001072	4	0.430	0.108
F	38	68.8	26.5	0.001470	55.5	0.001699	6	0.200	0.033
M	51	70.0	23.4	0.001167	62.7	0.001025	5	0.100	0.020
M	42	93.6	29.9	0.001776	79.8	0.001514	5	2.890	0.578
F	57	56.4	23.5	0.001744	54.0	0.001738	4	1.170	0.293
F	31	75.4	29.1	0.001455	60.2	0.001111	6	0.500	0.083
F	23	61.0	22.4	0.001497	56.0	0.002004	4	0.040	0.010
F	39	79.6	27.9	0.001138	73.5	0.001203	7	6.290	0.899
F	15	52.8	19.8	0.001144	52.6	0.001296	6	0.350	0.058
F	28	44.0	18.2	0.001538	45.0	0.001704	4	0.180	0.045
F	52	65.4	25.5	0.001254	56.2	0.001470	6	1.980	0.330
F	25	45.0	18.0	0.001736	47.6	0.002071	8	0.340	0.043
F	24	60.4	22.7	0.001350	60.4	0.001326	7	14.260	2.037
F	50	93.2	33.4	0.000764	87.6	0.000669	8	1.950	0.244
F	26	63.4	24.5	0.001289	56.0	0.001322	5	0.160	0.032
F	41	67.3	23.3	0.001394	64.1	0.001432	4	0.850	0.213
F	68	73.2	30.9	0.001321	66.6	0.001220	4	0.000	0.000
F	13	60.6	23.7	0.001442	53.0	0.001409	5	0.950	0.190
F	63	70.0	27.3	0.001229	62.8	0.001380	4	0.040	0.010
F	40	70.7	27.6	0.001213	63.6	0.001339	5	0.560	0.112
F	29	72.2	25.3	0.001690	64.0	0.001666	4	0.900	0.225
F	37	55.6	22.3	0.002071	50.6	0.001955	6	1.940	0.323
F	21	64.6	26.9	0.001446	65.0	0.001070	5	2.180	0.436

F	42	76.0	29.7	0.000979	55.9	0.001114	6	0.740	0.123
F	56	85.0	28.7	0.001240	76.6	0.001177	4	0.070	0.018
F	43	71.9	25.9	0.001168	65.4	0.001208	5	0.360	0.072
F	16	41.8	17.0	0.001553	44.8	0.002292	4	0.360	0.090
F	23	62.4	22.1	0.001749	59.4	0.001682	6	0.060	0.010
F	57	63.6	25.8	0.001489	54.8	0.001368	5	0.210	0.042
F	55	41.0	16.0	0.002744	43.0	0.003220	4	0.000	0.000
F	41	83.8	35.8	0.001070	71.2	0.001031	7	1.140	0.163
M	27	95.0	30.0	0.001866	81.4	0.001527	6	2.370	0.395
F	42	69.6	25.6	0.001364	67.0	0.001185	4	0.240	0.060
F	55	104.7	43.0	0.001076	87.9	0.001169	5	1.680	0.336
M	46	96.2	31.4	0.001274	85.0	0.001080	5	4.750	0.950
F	49	81.9	31.2	0.001410	67.5	0.001288	4	0.620	0.155
M	30	101.9	27.4	0.001760	92.0	0.001348	4	1.670	0.418
F	29	60.3	24.5	0.001905	55.1	0.001872	4	1.760	0.440
M	18	53.1	17.5	0.001569	54.4	0.001772	7	1.560	0.223
M	55	121.0	38.2	0.000538	104.2	0.000762	5	0.000	0.000
F	51	99.2	38.3	0.001233	90.0	0.001250	4	0.000	0.000
F	46	62.8	22.3	0.002196	59.6	0.001904	4	2.130	0.533
M	53	187.7	56.0	0.000970	136.8	0.000678	10	2.780	0.278
M	57	125.6	39.2	0.001017	100.0	0.000810	5	0.280	0.056

DF = degrees of freedom

Var= variance

Dev = deviance

Table S2. Summary of statistical analysis of studied sample

**Total deviance and variance (total SET)**			
**Number of patients**	**DF**	**Dev**	**Var**
374	1986	704.22	0.355
**Total deviance and variance (individual patient)**			
**Number of patients**	**DF**	**Dev**	**Var**
374	3.134	1.883	0.307

**Mean deviance and variance (calculated for healthy and some other pathological classes)**				
	**Number of patients**	**DF**	**Dev**	**Var**
**Healthy**	156	957	265.089	0.277
**Anorexia**	6	37	15.762	0.426
**Obesity**	127	779	274.208	0.352
**Dyslipidaemia**	77	472	126.968	0.269
**Hypertension**	72	441	111.573	0.253
**Diabetes**	50	307	60.479	0.197
**Thyroid**	45	276	79.212	0.287
**Tumours**	7	43	12.943	0.301

DF= degrees of freedom

Dev = deviance

Var= variance
